# Cross-cultural adaptation of the Patient-Doctor Relationship Questionnaire (PDRQ-9) in Brazil

**DOI:** 10.11606/S1518-8787.2018052000380

**Published:** 2018-07-13

**Authors:** Lucas Wollmann, Lisiane Hauser, Sotero Serrate Mengue, Milena Rodrigues Agostinho, Rudi Roman, Christina M Van Der Feltz-Cornelis, Erno Harzheim

**Affiliations:** I Universidade Federal do Rio Grande do Sul. Faculdade de Medicina. Programa de Pós-Graduação em Epidemiologia. Porto Alegre, RS, Brasil; II Tilburg University. Tilburg, Netherlands

**Keywords:** Physician-Patient Relations, Psychometrics, Translations, Interview, Surveys and Questionnaires, Validation Studies, Relações Médico-Paciente, Psicometria, Traduções, Entrevista, Inquéritos e Questionários, Estudos de Validação

## Abstract

**OBJECTIVE:**

To describe the process of cross-cultural adaptation of the Patient-Doctor Relationship Questionnaire (PDRQ-9), as well as compare the agreement between two different types of application.

**METHODS:**

This is a cross-sectional study with 133 adult users of a Primary Health Service in Porto Alegre, State of Rio Grande do Sul, Brazil. The PDRQ-9 was answered by the participants as a self-administered questionnaire and in an interview. The instrument was also validated by interview, using data from 628 participants of the *Mais Médicos* Program Evaluation Research, which is a cross-sectional study with a systematic sample of Primary Care Services in all regions of Brazil. We evaluated the semantic, conceptual, and item equivalence, as well as factor analysis and reliability.

**RESULTS:**

All items presented factor loading > 0.5 in the different methods of application and populations in the factor analysis. We found Cronbach’s alpha of 0.94 in the self-administered method. We found Cronbach’s alpha of 0.95 and 0.94 in the two different samples in the interview application. The use of PDRQ-9 with an interview or self-administered was considered equivalent.

**CONCLUSIONS:**

The cross-cultural adaptation of the PDRQ-9 in Brazil replicated the factorial structure found in the original study, with high internal consistency. The instrument can be used as a new dimension in the evaluation of the quality of health care in clinical research, in the evaluation of services and public health, in health management, and in professional training. Further studies can evaluate other properties of the instrument, as well as its behavior in different populations and contexts.

## RESUMO

**OBJETIVO:**

Descrever o processo de adaptação transcultural do *Patient-Doctor Relationship Questionnaire* (PDRQ-9), além de comparar a concordância entre duas diferentes formas de aplicação.

**MÉTODOS:**

Estudo transversal, com 133 usuários adultos de uma Unidade Básica de Saúde de Porto Alegre, RS. O PDRQ-9 foi respondido pelos participantes de maneira autoaplicada e por meio de entrevista. O instrumento também foi validado por entrevista, utilizando os dados de 628 participantes da Pesquisa de Avaliação do Programa Mais Médicos, um estudo transversal com amostra sistemática de Unidades Básicas de Saúde em todas as regiões do Brasil. Foram realizadas avaliações de equivalência semântica, conceitual e de itens, análise fatorial e avaliação da fidedignidade.

**RESULTADOS:**

Todos os itens apresentaram carga fatorial > 0,5 nos diferentes métodos de aplicação e populações na análise fatorial. Foi encontrado alfa de Cronbach de 0,94 no método autoaplicado. A aplicação por meio de entrevista encontrou alfa de Cronbach de 0,95 e 0,94 nas duas amostras diferentes. A utilização do PDRQ-9 por meio de entrevista ou de maneira autoaplicada foi considerada equivalente.

**CONCLUSÕES:**

A adaptação transcultural do PDRQ-9 no Brasil replicou a estrutura fatorial encontrada no estudo original, com alta consistência interna. O instrumento poderá ser utilizado como uma nova dimensão na avaliação da qualidade do cuidado em saúde em pesquisas clínicas, na avaliação de serviços e em saúde pública, na gestão em saúde e na formação profissional. Novos estudos poderão ampliar a avaliação de outras propriedades do instrumento, bem como seu comportamento em diferentes populações e contextos.

## INTRODUCTION

The doctor-patient relationship (DPR), in its historical context, depends on the medical situation and the social scene of each time^1^. Nonetheless, the perception of DPR as an important factor in the context of health care is a consolidated concept in multiple cultures^2^. The DPR involves components of verbal transfer of information associated with socioemotional aspects^3^. Nevertheless, there is no consensus regarding a universally accepted definition, because of the inherent complexity and subjectivity of the process. Operationally, DPR can be understood as a special type of human relationship that, either in terms of function or structure, is a component of care with the potential to affect health outcomes^4^ ^,^ ^5^. A satisfactorily developed DPR is associated with better symptom control, such as: pain, disability, anxiety^6^, weight loss, and blood pressure control^7^. In addition, it improves adherence to treatment^8^ and increases satisfaction with care^9^, which have a direct impact on the management of acute and chronic health problems^6^.

The DPR can be approached in different ways. It can be seen as the relationship of trust, therapeutic alliance, or empathy, developed between physician and patient^10^. It can also be treated as the ability of the physician to communicate and interact or the continuity of the care relationship^5^ ^,^ ^11^. Personal characteristics such as race, sex, socioeconomic status, and age, as well as the interpersonal and communication skills of the physician and their attire, are identified as factors with potential to affect the DPR^12^ ^–^ ^17^.

In the context of Primary Health Care (PHC), the DPR is inserted within longitudinality, which is one of the essential attributes of PHC defined by Starfield^18^. Interactions between professionals and patients contribute to the establishment of long-term relationships, which facilitate the effectiveness of PHC^18^. The DPR is also a key component of the person-centered approach^11^.

The DPR in the clinical setting is usually measured from the perception of patients^10^. The most frequently evaluated dimension refers to some type of alliance with descriptions such as: bond, goals, tasks, and collaboration. Other dimensions commonly evaluated are: trust, empathy, and communication skills^10^. Qualitative approaches are used as tools in the development of the conceptual structure of factors that define the doctor-patient relationship^5^. Quantitative assessments using validated scales are the most common procedures to measure the processes of doctor-patient interaction based on their advantages in terms of external validity and comparability of results^11^. The readness of scales in relation to application and analysis of results also favors their use in clinical or population studies and in the evaluation of professionals and health services.

We found no instruments that evaluate DPR in Brazil adapted to the scenario of outpatient medical practice. In addition, the Brazilian population of illiterates or functional illiterates reaches 17.6% of the persons aged 15 years or more, and this value can reach 27.1% in the Northeast region^19^. Therefore, we need a resource that can include these persons. The Patient-Doctor Relationship Questionnaire (PDRQ-9)^20^ is a questionnaire developed in the Netherlands in 2004. It has been translated and validated in the United States^21^, Germany^22^, Spain^23^, Turkey^24^, and Bangladesh^25^. It is considered adequate to the PHC scenario, because it is concise and easy to apply, and it has excellent reliability and internal consistency^10^. The objective of this study was to describe the cross-cultural adaptation process of the PDRQ-9 to the Brazilian context, as well as compare the agreement between two types of application.

## METHODS

We performed a cross-cultural adaptation according to the recommendations of the Consensus-based Standards for the Selection of Health Measurement Instruments (COSMIN Initiative)^26^, which is an international guideline for assessing the methodological quality of studies on the properties of health measurement instruments.

The PDRQ-9 is an instrument^20^ that evaluates DPR from the perspective of patients, focusing on their perception on the willingness to help and empathy of the physician. It was developed from a questionnaire that evaluates therapeutic alliance in psychotherapy. During the validation process of the original instrument, some items were added or modified, and the specific items of the psychotherapy scenario were removed, which resulted in a nine-item scale.

Each item of the instrument is a statement about different attributes of the DPR (help, time, trust, understanding, dedication, agreement, availability, contentment, and accessibility), which evaluate the relational and satisfaction aspects. The instrument was developed to be self-administered, and the patient should answer how much each statement is appropriate on a five-point Likert scale. In a population, the score of each item is calculated by the arithmetic mean of the answers of that item, and a general score is calculated by the arithmetic mean of the answers of the nine items.

Two samples were used in the process to evaluate the psychometric properties of the PDRQ-9. The sample of the main validation study (MVS) aimed to evaluate the instrument both when self-administered and when applied in an interview. The MVS was a cross-sectional study with 133 users in a Primary Care Service (PCS) in Porto Alegre, State of Rio Grande do Sul, Brazil. We used convenience sampling, stratified by sex and two age groups (18 to 59 years and ≥ 60 years). The strata were performed using data from a large PCS in Porto Alegre. Data collection took place between September and December 2016. Users were approached after medical consultation by trained interviewers. They should have four or more years of education and at least two appointments with that physician. They answered the self-administered PDRQ-9, deposited their answers in a ballot box, and then answered the same instrument in an interview. The patient did not know that they would have to answer the instrument again in an interview when invited to fill the self-administered questionnaire. In order to assess the stability of the scale over time, participants received the instrument again by letter or e-mail after two weeks in order to answer it at home.

We calculated the sample size of the MVS to test the equivalence between two paired means, according to the Bland-Altman procedure^27^, to evaluate the agreement of the PDRQ-9 that was self-administered and applied in an interview. We considered an overall instrument mean score of 4.1 and a standard deviation of 0.8^20^. As a reference for comparison between the methods of application, we used a Spanish study that applied the PDRQ-9 in an interview^23^. We stipulated an expected difference of 0.2, negligible difference of 0.3, correlation of 0.8, power of 0.8, and statistical significance of 0.05.

We also evaluated the instrument when applied in an interview using a sample of participants of the *Mais Médicos* Program Evaluation Research (PAPMM), which is a cross-sectional study with a systematic sample of Primary Care Services (PCS) throughout Brazil. The objective of this study was to evaluate the quality of the medical care offered to adult users of the Family Health Strategy (FHS) in Brazil. Cuban and Brazilian doctors of the *Mais Médicos* Program (PMM) were compared to Brazilian doctors who did not work with the PMM. In each PCS sampled, approximately twelve adult users (≥ 18 years) were approached, with at least two appointments with that physician by consecutive selection after appointment with a previously selected physician. These users answered several instruments to trained researchers, among them the PDRQ-9. Of the 6,200 users interviewed in the PAPMM, 10.0% of the participants were randomly selected for the evaluation of the properties of the PDRQ-9. This sub-sample was stratified by state, size of the city, number of FHS teams, and work category of the physician (part or not of the *Mais Médicos* Program). We did not include data of patients cared by Cuban doctors, since the purpose of the study was the cross-cultural adaptation to Brazil, including questions related to Brazilian Portuguese.

The instrument was selected by one of the authors (LW) after reviewing the literature on the subject. The face and content validity of the scale was evaluated based on national^28^ and international literature^10^ related to the attributes of DPR. The instrument was discussed by a committee of experts (two epidemiologists with experience in cross-cultural adaptation studies and three family physicians, all with English proficiency) to evaluate the conceptual and item adequacy in the Brazilian context.

Two translations were made from English to Portuguese by two independent translators who were native English speakers. Back translation to English was performed by another pair of independent translators, who were Brazilians fluent in English. Four pre-tests were performed with ten questionnaires in adult users, in the same PCS of the MVS. The objective of the questionnaire was explained to the participants, who were asked if they considered the statements comprehensible, and relevant results were discussed with the research team after each pre-test. Doubts were discussed with the author of the original instrument (CMVF).

We used factor analysis extraction with principal axis factoring to evaluate the validity related to the construct. We selected the items with factor loading above 0.30^29^. We evaluated the reliability of this instrument through internal consistency and stability over time. In order to evaluate the internal consistency of each component, we used the item-total correlation, considering as adequate the items with a value above 0.50, in addition to Cronbach’s alpha coefficient, considering an appropriate value if equal to or greater than 0.70^29^. Time stability and agreement analysis between the self-administered and interview methods were performed using the Bland-Altman procedure^27^, with Wilcoxon test, and intraclass correlation coefficient (ICC), which was considered appropriate if greater than 0.60^26^. The analyses were performed using the SPSS software, version 18.

This research was approved by the Research Ethics Committee of the *Hospital de Clínicas* of Porto Alegre in 2015 (CAAE 48653615.6.0000.5327) and by the Ethics Committees of all cities participating in the PAPMM that requested such approval. The information collected was kept confidential and the names of the participants were not disclosed. The data were presented grouped, keeping the confidentiality of each individual. All interviewees received a clear explanation of the objectives of the study. The participants signed the informed consent.

## RESULTS

The expert committee considered the instrument appropriate in relation to the face and content to be used in the Brazilian context. Translations and back translations were compared to each other and the original version, and the first version of the pre-test instrument was developed. The translation of the word “appropriate” into “*concordo*” (“agree”) in the answer options of the instrument (from 1 to 5, 1 being equivalent to “I do not agree” and 5 “I totally agree”) was suggested. This change was considered appropriate by the expert committee and approved by the author of the original instrument. In general, participants had a good understanding of the questionnaire. Different words and syntaxes were tested to improve understanding, keeping the original meaning of each item: item 6 – nature *versus* cause, symptoms *versus* medical symptoms; item 7 – speaking *versus* talking; item 8 – satisfied *versus* content; item 9 – have access *versus* easily accessible. At the end of the fourth pre-test, we reached the version to test the psychometric properties. There were no missing data in any of the questionnaires used in the MVS and PAPMM.

Table 1 shows the characterization of the participants of the two samples used in the evaluation of the PDRQ-9. The participants of the MVS were older, had higher education level, lower unemployment, and a lower proportion lived with a partner.

**Table 1:** Characterization of the sample of participants of the MVS and PAPMM. Brazil, 2016.

Variable	MVS (n = 133)		PAPMM (n = 628)	
	
	n	%	n	%
Sex				
Male	39	29.3	155	24.7
Female	94	70.7	473	75.3
Age^a^	55	18.0	48	17.1
Self-reported race				
White	111	83.5	213	33.9
Brown	15	11.3	319	50.8
Other (black, yellow, indigenous)	7	5.2	96	15.3
Do you live with a partner?				
Yes	70	52.6	404	64.3
No, but have lived before	51	38.3	154	24.5
Never lived	12	9.0	70	11.1
Work situation				
Working	59	44.4	239	38.1
Retired/benefit	52	39.1	191	30.4
Unemployed	22	16.5	198	31.5
Complete years of study^a^	11	3.7	7	4.6
Number of appointments with the physician in the last 12 months^b^	3	3.0	5	7.0

MVS: main validation study; PAPMM: *Mais Médicos* Program Evaluation Research

^a^ Data presented as mean (standard deviation).

^b^ Asymmetric data, presented as median (interquartile range).

Seventeen physicians were responsible for the care of the participants of the MVS. The mean age of physicians in this sample was 32 years and 70.6% were women; 29.4% were specialized in family medicine. The mean time of medical practice was 4.7 years, and they worked in the PCS for 2.3 years, on average. They had a mean weekly workload of 54 hours (considering all jobs) and cared for approximately 34 patients per week in the PCS. In the PAPMM, 52 physicians were responsible for the care of the participants, of whom two refused to provide their data. The mean age of physicians in this sample was 39 years and 50.0% were women; 72.0% were specialized in family medicine. The mean time of medical practice was 12.2 years, and they worked in the PCS of the research for 3.6 years, on average. They had a mean weekly workload of 60 hours, taking care of approximately 126 patients per week in the PCS of the research.

Factor loading of the self-administered PDRQ-9 in the population of the MVS was > 0.30 for all items, and item-total correlation was > 0.50 (Table 2).

**Table 2:** Mean score, standard deviation, factor loading for factorial validity, and item-total correlation of PDRQ-9 items by the self-administered method in MVS (n = 133). Porto Alegre, State of Rio Grande do Sul, Brazil, 2016.

Variable	Mean*	SD	Item-total correlation	Factor loading
My PCP helps me	4.6	0.7	0.70	0.73
My PCP has enough time for me	4.4	0.9	0.76	0.80
I trust my PCP	4.5	0.9	0.83	0.87
My PCP understands me	4.4	0.9	0.88	0.92
My PCP is dedicated to help me	4.6	0.8	0.85	0.88
My PCP and I agree about the nature of my medical symptoms	4.3	0.8	0.75	0.78
I can talk to my PCP	4.6	0.8	0.79	0.82
I feel content with my PCP’s treatment	4.5	0.8	0.85	0.88
I find my PCP easily accessible	4.1	1.1	0.52	0.53

MVS: main validation study; SD: standard deviation

* Variation of the score from 1 to 5.

We obtained an overall score of 4.45 (SD = 0.7) using the self-administered PDRQ-9. In the reliability assessment, we found Cronbach’s alpha of 0.94. The variance explained by the factor extracted was 65.3%.

Factor loading of the PDRQ-9 applied in an interview in the populations of the MVS and PAPMM was > 0.30, and item-total correlation was > 0.50 for all items (Table 3).

**Table 3:** Mean score, standard deviation, factor loading for factorial validity, and item-total correlation of PDRQ-9 items by the interview method in MVS (n = 133) and PAPMM (n = 628). Brazil, 2016.

Variable	Mean*		SD		Item-total correlation		Factor loading	
			
	MVS	PAPMM	MVS	PAPMM	MVS	PAPMM	MVS	PAPMM
My PCP helps me	4.4	3.3	0.8	0.9	0.86	0.70	0.90	0.73
My PCP has enough time for me	4.4	3.0	0.9	1.1	0.78	0.72	0.80	0.74
I trust my PCP	4.5	3.3	0.8	0.9	0.85	0.81	0.88	0.84
My PCP understands me	4.4	3.3	0.9	0.9	0.91	0.82	0.94	0.85
My PCP is dedicated to help me	4.6	3.2	0.8	0.9	0.83	0.80	0.86	0.83
My PCP and I agree about the nature of my medical symptoms	4.3	3.2	0.9	0.9	0.75	0.79	0.77	0.81
I can talk to my PCP	4.5	3.3	0.8	0.9	0.87	0.84	0.90	0.87
I feel content with my PCP’s treatment	4.5	3.3	0.8	1.0	0.86	0.81	0.89	0.84
I find my PCP easily accessible	4.1	3.1	1.1	1.1	0.54	0.76	0.55	0.78

MVS: main validation study; PAPMM: *Mais Médicos* Program Evaluation Research; SD: standard deviation

* Variation of the score from 1 to 5.

When evaluating the reliability of the PDRQ-9 applied in an interview in the MVS, we found a general score of 4.43 (SD = 0.7), with Cronbach’s alpha of 0.95, and variance explained by the extracted factor of 70.2%. In PAPMM, the overall score obtained was 3.23 (SD = 0.8), with Cronbach’s alpha of 0.94 and explained variance of 65.6%.

**Figure 1:**
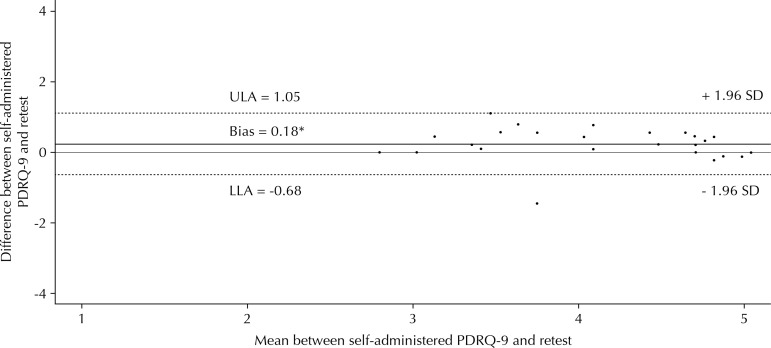
Bland-Altman scatter plot for time stability assessment of the PDRQ-9 in the MVS (n = 35). Porto Alegre, State of Rio Grande do Sul, Brazil, 2016.

ULA: upper limit of agreement; LLA: lower limit of agreement; SD: standard deviation; MVS: main validation study * Association with the Wilcoxon test (p = 0.004)

Thirty-five participants of the MVS completed the retest questionnaire sent after two weeks. There were no differences related to sex, race, age, education level, number of appointments, or score of the instrument between respondents and non-respondents of the retest. We found an intraclass correlation coefficient (ICC) of 0.96 (95%CI 0.94–0.98) between the retest and the self-administered instrument. The Bland-Altman scatter plot used to evaluate the time stability of the PDRQ-9 suggested a homogeneous distribution, with greater agreement for extreme values. The upper limit of agreement can be considered slightly enlarged (Figure 1).

**Figure 2:**
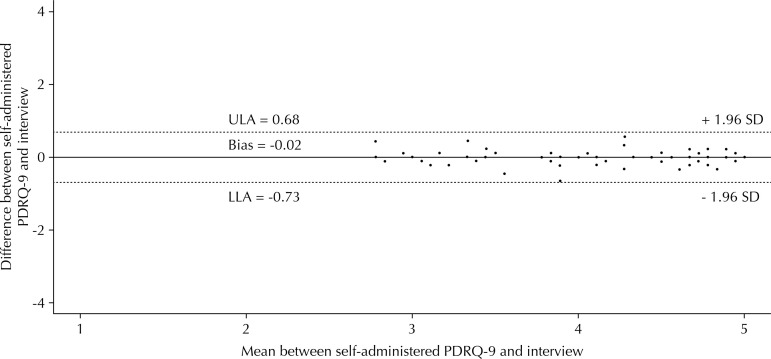
Bland-Altman scatter plot for analysis of agreement between the self-administered and interview methods in the MVS (n = 133). Porto Alegre, State of Rio Grande do Sul, Brazil, 2016.

ULA: upper limit of agreement; LLA: lower limit of agreement; SD: standard deviation; MVS: main validation study

The ICC was 0.94 (95%CI 0.93–0.95) in the assessment agreement between the self-administered and interview methods. The Bland-Altman scatter plot presented a homogeneous distribution, difference of means very close to zero, and narrow limits of agreement (Figure 2). We obtained p = 0.315 with Wilcoxon test.

## DISCUSSION

The cross-cultural adaptation of the PDRQ-9 replicated the one-dimensional structure observed in the original study^20^. From the results obtained, we could present the instrument to measure DPR considering the nine items, which showed appropriate performance for the validity and reliability measures evaluated (Box). Factor loading was high in all items and methods of application of the different samples, with a drop in the item related to access. This situation was also verified in the original validation study^20^. A possible explanation for this is that the semantic content of the term “access” carries meaning not only related to the availability of the physician, but also to the organization of the health service^18^. New studies with the instrument in services with easy and difficult access, controlling for this factor, may help in understanding the interference of accessibility related to the service in the evaluation of the DPR by users. Qualitative studies may help define a more appropriate sentence, in order to discriminate the availability of the professional.

**Box:** Final version of the PDRQ in Brazilian Portuguese.

Apresentation
*Eu vou ler pra você/Você vai ler nove frases sobre o relacionamento que você tem com o Dr._________________(MÉDICO DA PESSOA). Por favor, eu quero que você me diga/marque o quanto você concorda com cada uma dessas frases, de acordo com as seguintes alternativas:*

**Reply options**

*1 = Não concordo*
*2 = Concordo um pouco*
*3 = Concordo*
*4 = Concordo muito*
*5 = Concordo totalmente*

**Instrument items**

*Meu médico me ajuda.*
*Meu médico tem tempo suficiente para mim.*
*Eu confio no meu médico.*
*Meu médico me entende.*
*Meu médico se dedica a me ajudar.*
*Meu médico e eu concordamos sobre a natureza dos meus sintomas.*
*Eu consigo conversar com o meu médico.*
*Eu me sinto contente com o tratamento que o meu médico me oferece.*
*Eu acho fácil ter acesso ao meu médico.*

The high internal consistency verified in this study, either by the self-administered or interview method in the different populations (α = 0.94–0.95), can also be observed in the other evaluations of this instrument, such as the Dutch (α = 0.94)^20^, Germany (α = 0.95)^22^, Spanish (α = 0.95)^23^, American (α = 0.96)^21^, and Turkey ones (α = 0.91)^24^. The item-total correlation reached values considered appropriate in all populations and methods of application, for all items of the instrument. Different properties of the instrument have been tested in international studies, such as: convergent^21^ ^,^ ^22^, discriminant^20^ ^-^ ^22^, and confirmatory factor analysis^22^ ^,^ ^24^. As in the studies in other countries, the PDRQ-9 reached moderate or high scores in the evaluation of the DPR in Brazil. This finding is also verified in the use of different instruments that measure DPR^10^.

The application of the PDRQ-9 in the PAPMM allowed its cross-cultural adaptation, with a sample of participants from all regions of Brazil. These users were found in PHC services of the Brazilian Unified Health System, in their different types of organization and offer of care. In addition, we could include persons with great individual and social plurality. These factors add robustness to the presented results.

Although originally designed to be self-administered, the PDRQ-9 has already been validated in Spain for use through interviews^23^. However, it is the first time that the evaluation of the psychometric properties of the instrument is carried out in parallel for two different methods of application, which allowed us to verify the existence of differences between them. We found high correlation and the Bland-Altman procedure showed great agreement between the different types of application, which makes us consider that they are equivalent. The prospect of using PDRQ-9 in interviews allows the inclusion of illiterates and functional illiterates in later applications of this instrument.

To evaluate the stability of the scale over time, the response rate after two weeks was low (26.3%), which was also verified in the original PDRQ-9 validation study (33%)^20^. The answers of the participants presented excellent correlation. The bias found (0.18) was statistically significant but considered small. Greater agreement at extremes in relation to the center of the scatter plot suggests that persons who evaluate their physician with moderate scores present greater uncertainty in providing answers. The change in the answers of the participants after two weeks may be due to the effects of the recommendations or treatments prescribed over that period, as well as the fact that the instrument is answered outside the health service. A moderate correlation between test and retest was observed in the original study, the only one to perform an assessment of the stability over time, with Pearson coefficient of 0.61^20^.

This study presents limitations. We did not evaluate the time needed to answer the instrument. Therefore, we could not perform analyses related to learning bias or interference of factors such as education level. The participants of the MVS may have felt compelled to answer the instrument identically, as they had to answer the PDRQ-9 using two different methods in sequence, which may have underestimated the difference between the methods. The lack of knowledge of the participant on the fact that they would answer the instrument a second time minimizes this effect. On the other hand, the use of the ballot box reinforced that the goal in answering the instrument for the second time was not to remember what was already answered, but to provide a new authentic answer. The application of the instrument in the health service can lead to socially acceptable answers and overestimate the judgment of the persons towards their physicians. As in other studies, this was minimized by interviewers not tied to the service and the ensured anonymity of answers.

The cross-cultural adaptation of the PDRQ-9 to the Brazilian context allowed the availability of a concise and versatile instrument in the evaluation of the DPR, especially in the PHC scenario. It can be self-administered or applied in an interview. Further studies may evaluate other properties of the scale as well as their behavior in different population strata and specific contexts. The use of the PDRQ-9 will allow the inclusion of a new dimension of the quality of health care in clinical research, in the evaluation of services, in health management, in pay for performance, and in professional training.
